# Effective treatment of squamous cell carcinomas with ingenol mebutate gel in immunologically intact SKH1 mice

**DOI:** 10.1007/s00403-012-1270-0

**Published:** 2012-08-08

**Authors:** Sarah-Jane Cozzi, Thuy T. Le, Steven M. Ogbourne, Cini James, Andreas Suhrbier

**Affiliations:** 1 Queensland Institute of Medical Research, Post Office Royal Brisbane Hospital, Brisbane, QLD 4029 Australia; 2 Peplin Ltd, Brisbane, QLD Australia; 3 Griffith University, Nathan, QLD 4111 Australia; 4 Present Address: University of the Sunshine Coast, Maroochydore, QLD 4558 Australia

**Keywords:** Ingenol mebutate, Squamous cell carcinoma, Mouse model

## Abstract

Ingenol mebutate has recently been approved by the Federal Drug Administration (USA) as a topical treatment for actinic keratoses. Herein, we describe the efficacy of ingenol mebutate for the topical treatment of squamous cell carcinoma (SCC) using a wild-type mouse model (SKH1) and the UV-induced mouse SCC cell line, T7. Daily treatment for 2 days with 0.25 % ingenol mebutate gel produced a cure rate of 70 %, with 0 % for placebo gel. Electron microscopy revealed swelling of cancer cell mitochondria within 1 h, with disruption of the inner mitochondrial membranes evident at 6 h post treatment. Primary necrosis of cancer cells was clearly evident by 24 h. Treatment was associated with local haemorrhage and a prodigious neutrophil infiltrate, with anti-T7 antibodies also detected. This is the first report of the successful treatment of SCC tumours with ingenol mebutate gel in wild-type mice, and supports the view that ingenol mebutate induces primary necrosis and activates the immune system.

**Electronic supplementary material:**

The online version of this article (doi:10.1007/s00403-012-1270-0) contains supplementary material, which is available to authorized users.

## Introduction

Cutaneous squamous cell carcinoma (SCC) is the second most common human cancer after basal cell carcinoma, with >250,000 cases diagnosed in the USA each year. The usual treatment options include surgery, curettage and electrodesiccation, and radiation [2]. Topical imiquimod or fluorouracil may also be suitable for patients with small tumours in low-risk locations, who are unwilling or unsuitable to receive conventional treatments [10].

Following phase III studies [8], topical ingenol mebutate (ingenol-3-angelate, PEP005) was recently approved by the Federal Drug Administration (USA) for the treatment of actinic keratosis, a precursor of SCC. Ingenol mebutate was derived from the sap of *Euphorbia peplus*, and in a phase I/II clinical study, a complete clinical response was achieved in 2/4 SCCs treated topically with the sap from this plant [14]. Ingenol mebutate was also effective against inoculated murine SCC tumours (LK2 and PAM212) grown in nude mice (*Foxn1* ^*nu*^ or BALB/c^nu/nu^) [9, 12], with haemorrhage [9], neutrophils and antibody-dependent cellular cytotoxicity appearing to be important for relapse-free cure in these models [4]. In general, mouse studies have suggested that ingenol mebutate has a dual mechanism of action against tumours involving initial induction of primary necrosis [12] followed by immune-mediated clearance of residual tumour cells [17].

T cell deficient nude mice may not be ideal for testing treatments for SCCs, as SCCs are believed to be subject to immune control [20]. This contention may extend to the testing of ingenol mebutate treatment in nude mice [9, 12], as there is emerging evidence suggesting cross talk between neutrophils and T cells [13, 18], with ingenol mebutate treatment also shown to induce anti-cancer T cells and antibodies [4, 7]. Here we test ingenol mebutate in an immunologically intact mouse model, inbred SKH1 mice [5] inoculated with the T7 SCC line, derived from an SCC induced in these mice by chronic minimally erythemal ultraviolet irradiation [11].

## Materials and methods

### T7 cell line

T7 was provided by Dr G. Halliday (University of Sydney, NSW, Australia) and tested negative for mouse hepatitis virus, minute virus of mice, mouse parvovirus and rotavirus. Cells were maintained as described [11]. Exponentially growing cultures were trypsinised, washed once in growth medium, and resuspended in DMEM prior to inoculation. Electron microscopy of T7 tumours demonstrated the presence of desmosomes, confirming the epithelial origin of the T7 line (see Online Resource 1).

### Mice, tumour inoculation, treatment and monitoring

Inbred SKH1 hairless (*hr*/*hr*) mice (ARC, Perth, Australia) were bred at QIMR [5]. Mice (>4 weeks old) were inoculated (5 × 10^4^ T7 cells in 50 μl) by shallow s.c. injection on the back. Tumours were measured using digital callipers, and size were expressed as width × length. Tumours were treated topically (on days 4 and 5 post tumour inoculation) with 30 μl of 0.25 % (w/v) ingenol mebutate or placebo gel (supplied by LEO Pharma A/S) using a positive displacement pipette, with the gel then spread over the tumour site.

### Histology and electron microscopy

Histology and electron microscopy were undertaken as described [4, 12].

## Results

**Fig. 1:**
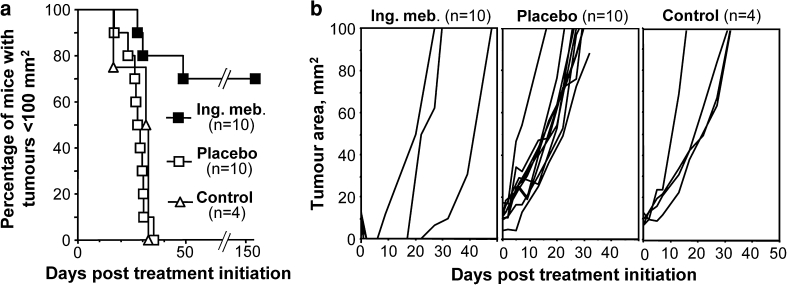
T7 SCC tumours growing on the back of inbred female SKH1 mice were treated daily for two days with 0.25 % ingenol mebutate or placebo gel. **a** Kaplan–Meier survival curves, with animals euthanased when the tumour reached 100 mm^2^. **b** Growth curves of the individual tumours described in **a**. The tumour sizes on day 0 were Placebo group mean 10.8 ± SD 3.4 mm^2^ (range 4–17.5 mm^2^), ingenol mebutate group 10.3 ± SD 2 mm^2^ (range 9–13.5 mm^2^)

Treatment of T7 SCC tumours growing in female mice with 0.25 % ingenol mebutate (once daily for two days) resulted in a 70 % cure rate, defined as no visible tumour after 150 days post treatment initiation (Fig. 1, logrank test, placebo vs ingenol mebutate, *p* = 0.0002). When the ingenol mebutate dose was reduced to 0.1 % in female mice, tumour growth was delayed significantly by ≈10 days (placebo vs ingenol mebutate, *p* = 0.0006), but the treatment failed to cure any of the tumours (data not shown).

Treatment of T7 tumours in male mice with 0.25 % ingenol mebutate resulted in a cure rate of 30 % (placebo vs ingenol mebutate *p* = 0.008, *n* = 10 per group) (data not shown). The difference between male and female mice treated with 0.25 % ingenol mebutate approached significance (*p* = 0.108).

**Fig. 2:**
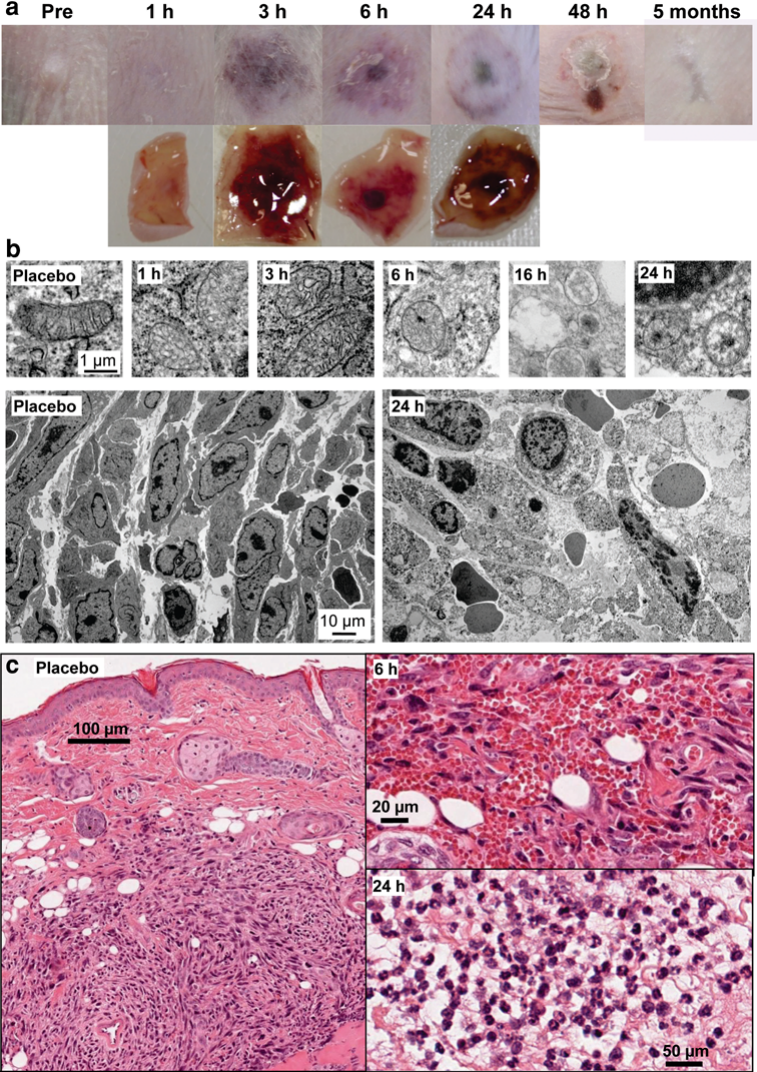
**a** Photographs of a T7 tumour site at various times post treatment with ingenol mebutate. *Top row*–pictures taken of the skin in the intact animal. *Bottom row*—pictures taken of the excised tumour site, with the skin turned over and photographed from the dermal side. **b** Electron micrographs. *Top row*–tumour cell mitochondria at the indicated times post ingenol mebutate treatment. *Bottom left*–T7 tumour cells after placebo treatment. *Bottom right*–T7 tumour cells 24 h after placebo treatment, showing clear signs of primary necrosis. **c** H&E staining of placebo treated tumour (*left*) and high magnification images of haemorrhage within the tumour 6 h post ingenol mebutate treatment (*top right*) and prolific neutrophil infiltrates 24 h post ingenol mebutate treatment (*bottom right*)

Within hours of ingenol mebutate treatment, clear signs of haemorrhage in and around the tumour site were evident. After 48 h an eschar formed over the tumour site, which resolved after 3–4 weeks, with a favourable cosmetic outcome evident after 2–3 months (Fig. 2a). Electron microscopy showed disruption of the inner membrane and cristae of the tumour mitochondria within hours of treatment, with complete loss of inner membrane structures evident at 16–24 h (Fig. 2b). At 24 h post treatment, primary necrosis of tumour cells was clearly seen (Fig. 2b).

Histology of placebo-treated tumours (which were indistinguishable from untreated tumours) illustrated their subcutaneous location ≈200–300 μm below the epidermis (Fig. 2c, left panel). Six hours after ingenol mebutate treatment, widespread haemorrhage was evident throughout the tumour (Fig. 2c, upper right panel) and the surrounding tissue (not shown). A neutrophil infiltrate was evident as early as 6 h post treatment (not shown), and by 24 h prodigious neutrophil infiltrates were seen in and around the tumour site (Fig. 2c, lower right panel).

## Discussion

Herein, we show that ingenol mebutate gel treatment (0.25 %) was effective at regressing subcutaneous SCCs using the T7/SKH1 mouse model. This study, together with previous clinical studies using *E. peplus* sap [14], supports further clinical development of ingenol mebutate for the treatment of SCCs in humans.

Ingenol mebutate gel treatment of SCCs in this model resulted in a rapid onset of haemorrhage in and around the tumour, disruption of tumour cell mitochondria and death of the tumour cells by primary necrosis. A prodigious infiltrate of neutrophils was observed, with post treatment SCC-specific IgG responses also apparent (see Online Resource 2), suggesting the activation of SCC-specific CD4 T cells. These observations are consistent with previous studies using murine SCC lines grown in nude mice and mouse B16 melanomas grown in C57/BL6 mice [4, 7, 9, 12], and support the view that ingenol mebutate gel treatment kills tumour cells by inducing primary necrosis with both the innate and adaptive immune systems being activated.

The ingenol mebutate concentration in the gel (0.25 %) required to cure T7 tumours was higher than the 0.05 % being used to treat actinic keratoses in humans [8]; however, 0.25 % ingenol mebutate gel has been tested for the treatment of superficial basal cell carcinomas in a small number of patients [6]. LK2 tumours in nude mice were effectively treated with 0.1 % ingenol mebutate given daily for 3 days [4]. The reason for the lower dose requirement in the LK2/nude model is unclear, with no overt differences in neutrophil infiltration apparent in the LK2/nude and SKH1/T7 models. The LD_90_ values for in vitro treatment of LK2 and T7 cells with ingenol mebutate were also both ≈100 μg/ml (data not shown). The differences may reflect increased drug penetration through nude mouse skin [16] or other differences in skin biology, rather than be related to immunological differences between the mouse strains. The possible difference (*p* = 0.108) in cure rates for male and female SKH1 mice may similarly reflect increased drug penetration through female mouse skin [3]. Gender differences in the thickness of different skin layers have been reported for SKH1 and other mice [1, 15], and in our experience SKH1 male skin is tougher and harder to inject than female skin. Such skin differences in mice may influence drug penetration, but may also affect other factors such as the consistency of shallow s.c. injections of tumour cells and/or local dissemination of the tumours. It should also be noted that there were no gender differences in the efficacy of ingenol mebutate in the treatment of actinic keratoses in humans [8].

Although SCCs are believed to be subject to immune control [20], there is a paucity of SCC models in immunologically intact mice [19]. We also investigated the 13.1 SCC line derived from C3H/HeN mice [11] and confirmed its epithelial origin using electron microscopy (data not shown). However, variable spontaneous regression rates (0–60 %) and variable growth rates (10–60 days to reach 100 mm^2^) made this model difficult to use. In contrast, the T7 SCC model provides a convenient and a reliable model for testing interventions in immunologically intact mice; however, the model does require an inbred SKH1 colony, as these mice are no longer commercially available.

## Electronic supplementary material

Below is the link to the electronic supplementary material.

Supplementary material 1 (PDF 262 kb)
