# Supplementary material for: Effective treatment of squamous cell carcinomas with ingenol mebutate gel in immunologically intact SKH1 mice
Source: Arch Dermatol Res. 2012 Aug 8;305(1):79–83. doi: 10.1007/s00403-012-1270-0 (PMC3535356; doi:10.1007/s00403-012-1270-0)
Supplement: Supplementary file 1 — Supplementary material 1 (PDF 262 kb) [file 403_2012_1270_MOESM1_ESM.pdf]

Online Resource

Effective treatment of squamous cell carcinomas with ingenol mebutate gel in immunologically intact SKH1 mice. Arch Derm Res. Sarah-Jane Cozzi, Thuy T. Le, Steven M. Ogbourne, Cini James, Andreas Suhrbier. Queensland Institute of Medical Research. andreasS@qimr.edu.au

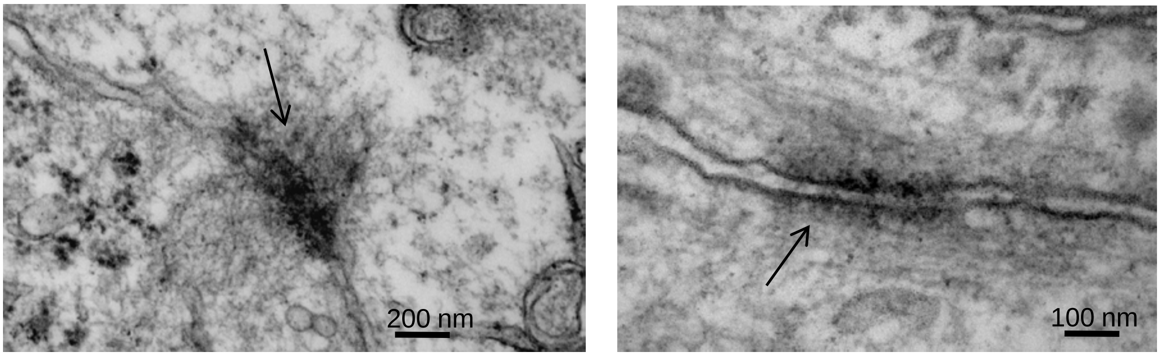

Online Resource 1. Transmission electron microscopy of a T7 tumour (day 20 post inoculation). Arrows indicate desmosomes and associated keratin filaments, indicating the epithelial origin of the tumour (Erlandson, R..A. 1981. Diagnostic transmission electron microscopy of human tumors: the interpretation of submicroscopic structures in human neoplastic cells New York: MASSON Publishing USA, Inc.).

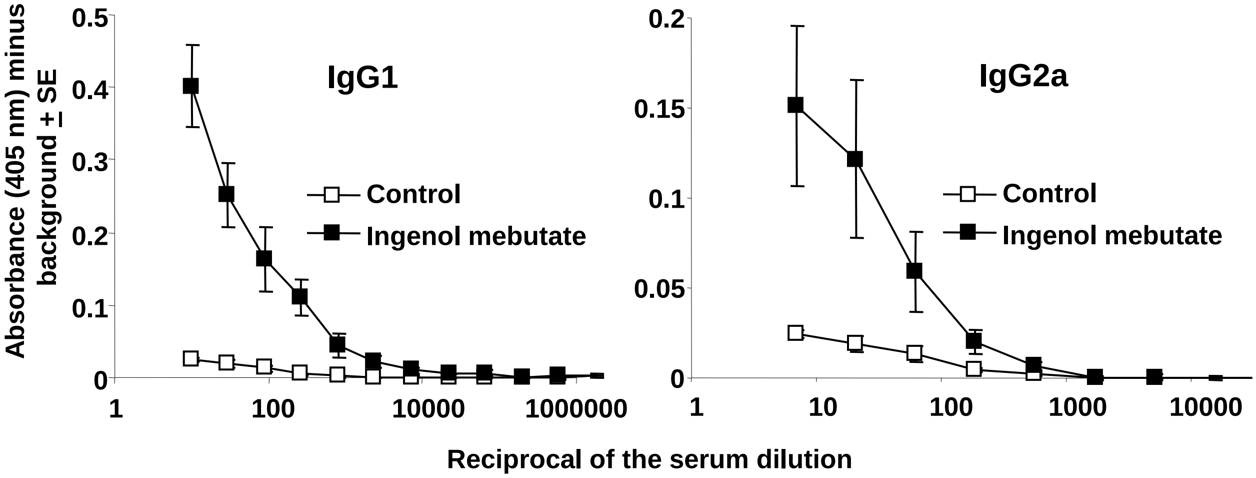

Online Resource 2. Anti-T7 IgG responses**.** ELISA assays were performed on serum obtained from female mice that had T7 s.c. tumours cured by topical treatment with 0.25% ingenol mebutate gel 100 days previously (Ingenol mebutate). Control mice are naïve SKH1 mice. All absorbances at serum dilution lower than 1/10000 (IgG1) and 1/1000 (IgG2a) were significantly different between the two groups (Mann Whitney U, $p<0.03$). Blood (~50-100 μl) was taken by tail bleeding into 0.8 ml MiniCollect® Z Serum Separator tubes (Greiner) . The ELISA plates with T7 lysate as antigen were prepared, and the ELISA assays were performed, as described previously (Challacombe et al., J Immunol. 2006, 177: 8123–8132).
